# Supplementary material for: STANDARD M10 SARS-CoV-2 Assay for Rapid Detection of SARS-CoV-2: Comparison of Four Real-Time PCR Assays
Source: Diagnostics (Basel). 2022 Aug 18;12(8):1998. doi: 10.3390/diagnostics12081998 (PMC9406901; doi:10.3390/diagnostics12081998)
Supplement: Supplementary file 1 [file diagnostics-12-01998-s001.zip › diagnostics-1728472-supplementary.pdf]

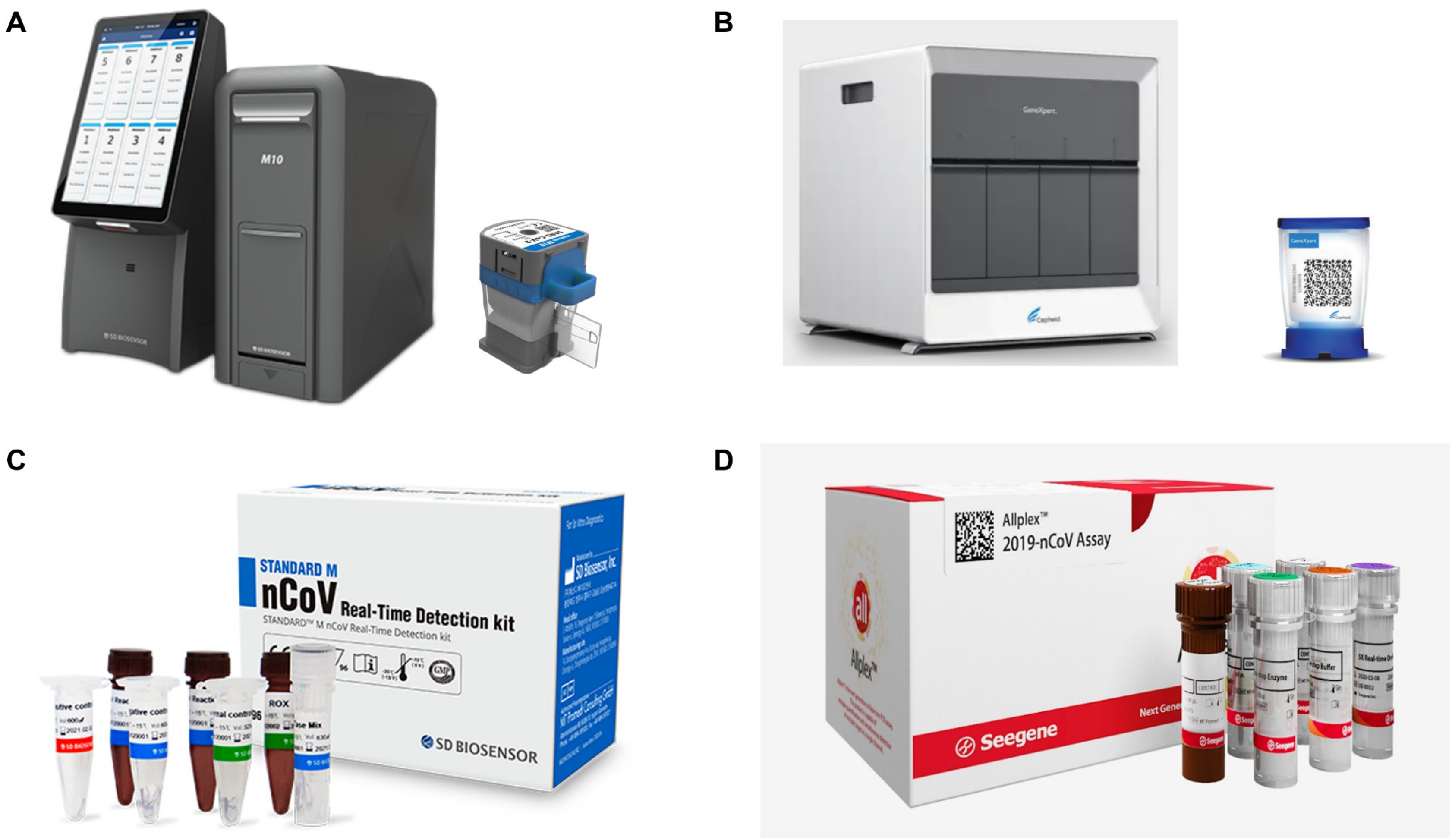

Figure S1. Photographs of two rapid RT-qPCR assays and conventional RT-qPCR assays. (**a**) STAMDARD M10 SARS-CoV-2 (SD Biosensor); (**b**) Xpert Xpress SARS-CoV-2 (Cepheid); (**c**) STANDARD M nCoV real-time detection kit (SD Biosensor); (**d**) Allplex 2019-nCoV (SARS-CoV-2) assay (Seegene).

Table S1. Ct values of nine samples with inconclusive results [1].

No.	Age (years)	Sex	STANDARD M10 SARS-CoV-2 (E)	STANDARD M10 SARS-CoV-2 (ORF1ab)	Xpert Xpress SARS-CoV-2 (E)	Xpert Xpress SARS-CoV-2 (N)	STANDARD M nCoV (E)	STANDARD M nCoV (ORF1ab)	Allplex SARS-CoV-2 (E)	Allplex SARS-CoV-2 (RdRP)	Allplex SARS-CoV-2 (N)
1	28	M	29.42	29.56	29.9	33.1	34.11	36.68	38.03	Negative	Negative
2	53	F	Negative	Negative	42.1	44.4	Negative	33.29	36.43	37.25	37.96
3	95	F	Negative	Negative	38.7	Negative	Negative	33.12	38.05	39.42	38.22
4	28	M	33.69	Negative	Negative	Negative	33.01	33.89	36.92	Negative	37.01
5	42	M	Negative	32.48	32.5	34.3	32.19	32.00	33.30	34.83	31.28
6	28	F	32.75	Negative	32.2	32.8	32.20	34.54	35.58	37.48	33.30
7	40	F	Negative	34.31	36.1	38.5	31.48	32.11	35.36	35.26	36.32
8	25	F	Negative	Negative	Negative	39.3	35.50	33.47	36.96	38.72	35.90
9	78	M	Negative	Negative	37.6	Negative	Negative	Negative	Negative	Negative	Negative

[1] Samples showing partial positivity among multiple target genes of each assay were designated as inconclusive results. Ct, cycle threshold.
